# Perspective on the Therapeutics of Anti-Snake Venom

**DOI:** 10.3390/molecules24183276

**Published:** 2019-09-09

**Authors:** Isabel Gómez-Betancur, Vedanjali Gogineni, Andrea Salazar-Ospina, Francisco León

**Affiliations:** 1 Ophidism-Scorpionism Program, Faculty of Pharmaceutical and Food Sciences, University of Antioquia UdeA, Medellín 1226, Colombia; 2 Analytical Department, Cambrex Pharmaceuticals, Charles City, IA 50616, USA; 3 Research group in Pharmacy Regency Technology, Faculty of Pharmaceutical and Food Sciences University of Antioquia UdeA, Medellín 1226, Colombia; 4 College of Pharmacy, University of Florida, Gainesville, FL 32610, USA

**Keywords:** Anti-venom, medicinal plants, plant constituents, snakebite treatment, snake venom

## Abstract

Snakebite envenomation is a life-threatening disease that was recently re-included as a neglected tropical disease (NTD), affecting millions of people in tropical and subtropical areas of the world. Improvement in the therapeutic approaches to envenomation is required to palliate the morbidity and mortality effects of this NTD. The specific therapeutic treatment for this NTD uses snake antivenom immunoglobulins. Unfortunately, access to these vital drugs is limited, principally due to their cost. Different ethnic groups in the affected regions have achieved notable success in treatment for centuries using natural sources, especially plants, to mitigate the effects of snake envenomation. The ethnopharmacological approach is essential to identify the potential metabolites or derivatives needed to treat this important NTD. Here, the authors describe specific therapeutic snakebite envenomation treatments and conduct a review on different strategies to identify the potential agents that can mitigate the effects of the venoms. The study also covers an increased number of literature reports on the ability of natural sources, particularly plants, to treat snakebites, along with their mechanisms, drawbacks and future perspectives.

## 1. Introduction

Snakebite envenomation is a grave public health issue in many regions of the planet, particularly in Africa, Asia, Central and South America, and Oceania. Snakebite envenomation is a neglected tropical disease that kills more than 100,000 people and maims more than 400,000 people every year. Members involved in public health contemplate that the neglected tropical diseases (NTDs) that affect human lives in developing countries are generally tropical infectious diseases, such as guinea worm, leishmaniasis, dengue, onchocerciasis, Chagas disease, and leprosy. The World Health Organization (WHO), together with the support of drug manufacturers and special interest groups, initiated a coordinated effort to address several of these diseases in 2005. Subsequently, a historical report on these problems was published in 2010, and consequently a strategic plan for eradication and control emerged. Today, the WHO lists 17 NTDs on its website as targets for eradication. On 27 May 2013, the WHO World Health Assembly adopted resolution EB132.R7, urging the 194 member states to expand their programs to prevent, control, eliminate, and eradicate all the 17 NTDs [1]. Although the WHO added snakebite envenomation to its official NTD list in March 2009, it was later quietly removed along with podoconiosis and strongyloidiasis and added to a separate list of what is called “other forgotten conditions”; a list that is not included in any of the WHO plans to eradicate NTDs, and is not even mentioned in their reports for 2010 and 2013 [2]. Fortunately, snakebites were re-included in category A of neglected tropical diseases (NTDs) in June 2017 and are recognized by the WHO [3]. This recognition is important and necessary to change the reality of this neglected tropical disease that continues to inflict a high cost of suffering and chronic disability in many of the poorest and most marginalized communities in the world.

It has been difficult to identify the exact incidence due to inadequate health statistics and the fact that some patients do not pursue medical care. In 2008, global annual incidence was estimated to be between 1.8 to 5.4 million bites [4]. According to the WHO, recent estimates suggested around 80–140,000 deaths/year, 400,000 amputations, and other long-lasting incapacities and disabilities caused due to snakebite envenomation [5]. Snakes with major clinical importance belong to the families Elapidae (African and Asian cobras, Asian kraits, African mambas, American coral snakes, Australian and New Guinean venomous snakes, and sea snakes) and Viperidae (Old World vipers, American rattlesnakes and pit vipers, and Asian pit vipers). An extended list is found in the WHO guidelines for the production, control, and regulation of snakebite antivenom immunoglobulins [6]. 

Envenomation is the result of the injection of a highly specialized toxic secretion, called venom, by a venomous snake into a human, usually in accidental situations [7]. Venom is injected through the snake’s fangs, which are teeth connected via a duct to a venom gland. The composition of snake venom shows high complexity and diversity, resulting in variable biochemical and toxicological profiles that determine a wide range of clinical manifestations. Snake venoms contain enzymes, including phospholipases A2, metalloproteases, serine proteases, L-amino acid oxidases, nucleotidases, and hyaluronidases [7,8]. The pathophysiology of snake envenomation involves a complex series of events that depend on the combined actions of these venom components. Toxins of the *Bothrops* snake venom mainly affect the muscle tissues along with blood clotting. Symptoms include pain, swelling, and bleeding (at the site of the bite and the mucous membranes), and in cases where complications can occur, blisters, gangrene, shock, and acute renal failure are observed. In other types of envenomation, such as from the genus *Crotalus* venom, the nervous system (neurotoxic) and muscles are affected along with clotting, and in severe cases acute renal failure and death may occur [9]. However, the presence and concentration of snake venom can vary depending on the geographic distribution, age, sex, diet, size, and season, among others [10]. Local tissue damage effects, such as hemorrhage, myonecrosis, and edema, are among the most dramatic effects of envenomation.

Ophidic accidents are a serious problem in public health due to the resulting high morbidity and mortality rates. The only specific treatment available is antivenom. Therefore, the search for complementary alternatives for snakebite treatment is very important and necessary. In order to develop alternatives to current therapies, researchers have been looking for bioactive compounds isolated from plant extracts with different properties such as analgesic, anti-myotoxic, anti-hemorrhagic, and anti-inflammatory effects for many years. In recent years, studies have been published that have provided pharmacological evidence regarding the benefits of various extracts and compounds isolated from different plant species against the local and systemic effects induced by a wide range of snake venoms, including lethality [11,12,13]. In this context, this review has aimed to provide an up-to-date description of the bioactive compounds that are isolated from various natural sources and tested as potential anti-ophidic agents and to unify the information and relevant data to help understand the diversity of these bioactive compounds, their actions on snake venom, and the prospects for future applications against venomous toxins. Additionally, a brief discussion on the open opportunities of using snake venoms as therapeutic agents is included.

## 2. Methodology

An extensive review of the literature was conducted, which came from different scientific sources, such as PubMed (https://www.ncbi.nlm.nih.gov/pubmed), Science Direct (http://www.sciencedirect.com/), Scopus (https://www.scopus.com/), Web of Science (http://www.webofknowledge.com/), Scientific Electronic Library Online (SciELO) (http://www.scielo.org/), Scifinder, and Google Scholar (https://scholar.google.com). The study databases included original articles published in peer-reviewed journals, as well as books, theses, dissertations, patents, and other reports covering information related to plants used in the treatment of snakebite envenomation (ethnopharmacological studies, original articles, or comments). The data and summaries of the complete articles were considered, selecting information in English, Portuguese, and Spanish. Publications with ethnobotanical studies, bioactive substances, or bio-guided pharmacological studies were analyzed in the search for therapeutic alternatives for the treatment of snakebites.

## 3. Current Information in the Design of New Antivenoms

Currently, the only accepted treatment for snakebite envenomation involves intravenous administration of conventional antivenoms comprising antibodies or antibody fragments derived from the plasma of large mammals (generally horses, but also sheep, goats, or rabbits) that have been previously immunized with non-lethal venomous doses [14,15]. Hyperimmunized animals produce antibodies against the venom proteins and serum is extracted from their blood for the treatment of envenomation [6,16]. Conventional serum therapy aims to bind and neutralize the snake venom proteins [17]. It is a fact that the antivenom allows the body to try to reverse the damage caused by the venom. However, it is known that such therapy can cause problems related to different antivenom characteristics, such as:

- Immediate hypersensitivity reaction to the alien immunoglobulins, including anaphylactic and pyrogenic reactions such as chills, rigor, headache, and tachycardia. Delayed antivenom reactions or serum sickness is observed after 8 to 12 days of treatment; these are characterized by cutaneous eruptions, fever, and allergies, among other effects [18];
- Limited efficacy of antivenom therapy to protect the affected organ/s against immediate local tissue damage and low stability;
- Ineffectiveness of the antivenom due to significant geographic variation in the composition of the venom;
- Antigenic reactivity due to the taxonomic diversity of the snakes;
- Improper use of the antivenom due to incorrect medical management, which contributes to a high incidence of adverse reactions, a low toxin neutralizing potency, or both.

Manufacture of antidotes from hyperimmunized animals is another problem that affects the access and use of antidotes in rural populations, mainly in developing countries. There is a deficiency in the availability of antidotes and lack of training in the clinical staff, worsening the consequences of the ophidian accidents and leading to increased incidence of death. These factors, together with distribution problems, unattractive profit margins, and high costs in developing and developed countries, reduce the viability and the interest of the pharmaceutical industries in the manufacture of antivenom serums [19]. There has been a decline in the number of producers in both private and public sectors. Chippaux provides three reasons for this dramatic reduction:

- The instability in the antivenom market;
- Little financial incentive for pharmacists and health centers to sell antivenom due to the low profit margins;
- Lack of comprehensive data on how many doses of antivenom are required and where they should be distributed [20].

Moreover, we must consider the supplementary therapeutic actions that a snakebite patient may require for effective treatment, such as the use of extra drugs, wound care services, reconstructive surgery, and rehabilitation therapy, all of which increase the total cost associated with this NTD.

Recombinant antivenoms with oligoclonal mixes of human monoclonal antibodies are the next-generation therapy for improved treatment of snakebites. A cocktail of human antibodies is needed for efficacious and high-quality biosynthetic oligoclonal antibodies (BOA) for the treatment of snakebites. BOA cocktails include all or most of the significant toxins that are needed for the pathophysiology of a snakebite. These toxins range from 20 to in excess of 40 neutralizing antibodies together with carefully selected human recombinant antibodies for a specific number of snake venoms. Depending on the specificity and number of monoclonal antibodies, BOAs can be monovalent or polyvalent. The general idea of the combination of small molecule inhibitors together with a BOA cocktail is that it could lead to improved neutralization of toxins in distal tissue by improving the pharmacokinetics [21]. With the increased bioinformatic tools and the availability of protein structural databases, several metabolites isolated from different sources, such as plants, bacteria, fungi, and other synthetic processes, have been evaluated for their ability to inhibit enzymes found in snake venoms [22]. Small molecule enzyme inhibitors could provide several potential advantages; for example, serving as adjuvants and reducing the effects of intoxication while the patient is being treated in a health care center, which would increase the recovery time, thereby improving the treatment window. Furthermore, since many of these small molecule inhibitors have been evaluated for their toxicity in human receptors, they have already proven to be safe enough to be used in the treatment of snakebite envenomation. 

Another approach for the development of snake antivenoms includes independent venom immunization techniques. There are four different strategies involved in this approach. The first strategy includes the injection of chemically synthesized epitopes of toxins, where bioinformatic software is used for the prediction of epitopes or epitope mapping studies. The second strategy involves not only the toxin epitopes but also the use of full-length recombinant or synthetic toxins as immunogens. In the third strategy, molecules with non-identical amino acid sequences that mimic the structure of the toxin epitopes, called mimotopes, are used. The final strategy involves the use of DNA, avoiding recombinant expression or chemical synthesis [14,23].

## 4. Current Drugs for the Treatment of Snakebites in the United States

The country most-affected by snakebites is India, with 46,000 deaths/year and around 4.1 cases of snakebite per 100,000 inhabitants [24]. According to the Centers for Disease Control and Prevention (CDC), an estimated 7000 to 8000 snakebites occur annually in the United States (CDC 2018). The 2017 annual report of the American Association of Poison Control Centers (AAPCC) showed around 7000 snakebites. Copperheads (2035), Crotalids, (1028), and rattlesnakes (753) were reported as the predominant species involved in envenomation; however, death is a rare outcome and only 5–10 deaths were reported [25]. Commercial antivenoms comprise the polyvalent and monovalent immunoglobulins, which are employed for a group of snake species or a single snake species, respectively [26]. In the United States, there are three Food and Drug Administration (FDA) approved drugs to treat snakebites:

- Antivenin^®^ Wyeth (equine), approved to treat envenomation from the American coral (Micrurus fulvius) [27];
- Crofab^®^ Crotalidae polyvalent immune FAB (ovine), approved in 2000 for snakebite envenomation from multiple species, including Crotalus atrox (western diamondback rattlesnake), Crotalus adamanteus (eastern diamondback rattlesnake), Crotalus scutulatus (Mojave rattlesnake), Agkistrodon contortrix (copperhead), and Agkistrodon piscivorus (cottonmouth or water moccasin) [28];
- Anavip^®^ Crotalidae Immune F(ab’)2 (equine), approved in 2015 to treat envenomation from the rattlesnakes Crotalus durissus and Bothrops asper [29].

Current antibody production faces challenges during the immunization of the animal (equine or ovine), leading to the production of a huge number of antibodies that are not related to the snake venom. Around 70% of the immunoglobulins obtained do not act directly against venom toxins [26]. Despite the abovementioned facts, this is the only FDA approved therapy to treat snake venom.

In many countries, the production and manufacture of antivenoms are mainly managed by governmental agencies or laboratories that are directly linked to the local national health authorities [30]. For example, since 1930 the Commonwealth Serum Laboratories (CSL) in the Seqirus division have produced mono- and polyvalent snake antivenoms for the most dangerous snakes in Australia, including black snake (*Pseudechis* genus), brown snake (*Pseudonaja* genus), death adder (*Acanthophis* genus), sea snake (*Hydrophis spiralis*), taipan (*Oxyuranus* genus), and tiger snake (*Notechis scutatus*). CSL provides the antivenoms for Australia’s universal health care system, which distributes these antivenoms within its territory. Despite the high number of dangerous snakes in Australia, the average number of deaths from snake envenomation is only 4 per year [31].

## 5. Folkloric Medicine in the Treatment of Snake Envenomation 

**Figure 1:**
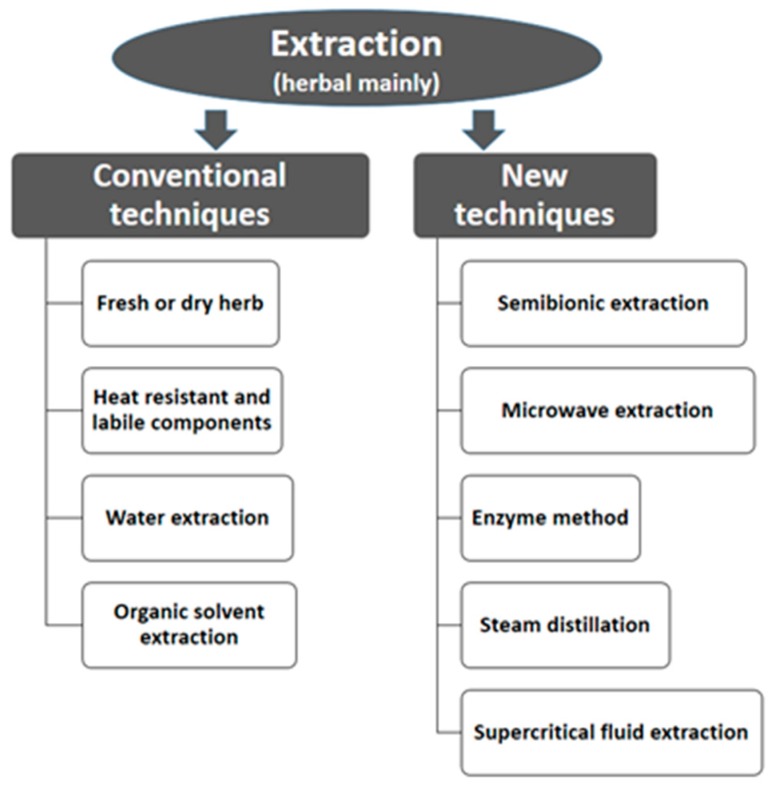
Current extraction techniques for herbal medicines.

In traditional medicine, herbal preparations were mainly used as decoctions, and this dosage form is still used in Asian, African, and Central and South American countries. The disadvantages of the herbal decoction include drawbacks in terms of use, unstable composition, and stability problems. Difficulties of decoctions could be overcome using plant extracts, which contain hundreds of active ingredients that can be potentially useful in the development of therapeutic agents. In addition, there are several other conventional and current techniques used to extract compounds from their natural sources (Figure 1). The identification and isolation of phytochemical groups from natural sources are, therefore, crucial in the drug discovery paradigm. It has been estimated that there are at least 15 major chemical groups in all the natural sources (flavonoids, alkaloids, glucosides, glycosides, volatile oils, resins, phytochromes, organic acids, amino acids, tannins, proteins, enzymes, trace elements, polysaccharides, and mineral salts among others). For example, flavones include more than 9000 known structures [32]. Alkaloids are a major class of compounds; thousands of alkaloids have been isolated. From which hundreds are being used clinically. Secondary metabolites from natural sources with hemostatic, antidiarrheal, antiulcer, antimicrobial, antiviral, wound healing, antitumor, anti-inflammatory, and anti-oxidant properties can be a foundation for therapeutic antivenom drugs [32].

The process of developing typical drugs or even a phytotherapeutic from herbal sources is a long process that includes at least four stages:

- The isolation or derivatization of bioactive substances from natural sources;
- The evaluation of safety and efficacy using pharmacological methods;
- Evaluation of safety and efficacy by conventional pharmacological methods (pharmacodynamics, toxicology, and pharmacokinetics);
- Regulatory approval of the therapeutic agent to be used in the market and in post-marketing supervision, as well as pharmacovigilance.

Studies showed that secondary metabolites isolated mainly from plants can clearly affect human homeostasis [33] by becoming the basis for production of many phytotherapeutics [34,35] and in the design of new medicines [36,37]. Currently, more than 55% of medicinal compounds are derived from natural products. Furthermore, 60% of the current anticancer compounds and 75% of the medicines used in the treatment of infectious diseases are either natural products or products derived from or inspired by natural sources, or use their pharmacophore as a model [38].

Plant extracts, fractions, and isolates have demonstrated the inhibitory activity of snake venoms, including their purified toxins. These inhibitors not only reduce the local tissue damage but also delay the easy diffusion of systemic toxins, and therefore, increase the survival time of the patient. The continuity of the studies on the mechanism of action and the safety of these molecules will reveal their potential use in the development of new therapies for snakebites. Several lists of medicinal plant species with activity against snake venom have been published, adding more than a thousand species that are used in folk medicine around the world [12,39].

Many indigenous and farmer communities make use of the resources available to them (usually plants) as an alternative to antivenom therapy, using extracts, baths, or infusions of medicinal plants in an effort to treat or minimize venom effects, such as hemorrhage and edema [40]. Clearly, ethnobotanical studies have been able to identify medicinal plants and active compounds that inhibit the action of snake venom. This knowledge has been used for the development of alternative therapies to treat the effects of snakebite envenomation. Although it is known that many plants are active against various effects of snakebites, few studies have been conducted to investigate the validity of these results under controlled conditions in both in vitro and in vivo models in order to demonstrate if there is any protective effect using simple molecules or prepared mixtures simulating traditional formulas [40]. For instance, ethnobotanical studies on the use of Costa Rican tropical plants for the treatment of snakebites have shown interesting results, identifying to a significant number of plants from different families that could potentially inhibit the toxic effects of *B. asper* venom. In fact, the organic and aqueous extracts of several plants have shown total inhibition of the hemorrhagic effects in rodent models after intradermal injections of the venom or a mixture of venom-extracts [41]. The anti-hemorrhagic activity can be attributed to the presence of active principles in some plants, such as 4-nerolidylcatechol and edunol [42,43,44,45], as well as flavonoids, which can chelate the zinc atoms involved in the catalytic activity of the venom’s hemorrhagic metalloproteinases, thereby neutralizing the hemorrhagic activity [41]. Amui and collaborators provided an innovative solution to systematize the information on medicinal plants with antivenom properties. They created a large public database that allows fast and reliable searches for information. The plant antivenom database has three types of information: medicinal plants with anti-venom properties, amino acid sequences of venom toxins, and enzymes of this theme. This computational tool displays relationships between data through different search schemes, providing a helpful resource for researchers in this field [46].

It is known that medicinal plants used against snakebites are found throughout the world, especially in tropical or subtropical regions of the Asian, American, and African continents. The need for alternative or complementary therapies to treat envenomation from snakebites is triggered due to the biodiversity related factors of the flora of these regions, as well as the difficulties in accessing adequate health services. According to Table 1, numerous studies have published several lists of medicinal plant species with activity against snake venom, adding more than 700 species used in folk medicine around the world [11,13,39]. These studies showed many ethnopharmacological investigations, the results of which demonstrated proven activities of medicinal plants used for the treatment of snakebites. A summary of some of these studies is shown in Table 1.

## 6. Mechanisms of Action of Antivenoms Derived from Herbs

The antigen–antibody reaction is the basic mechanism for the neutralization of snake venom by antivenoms. For small molecules, there are some hypotheses proposed on how those small compounds neutralize the toxic components of the venom. Several literature reports indicate the mechanism of inactivation; for example, through precipitation or inactivation of proteins [76], inactivation or enzyme inhibition [77], chelation [41], adjuvant action [78], antioxidant activity [79], or a combination of these activities. Among these, protein precipitation and enzyme inhibition are the most accepted [40].

### 6.1. Protein Precipitation 

Several secondary metabolites with protein binding properties against snake envenomation include flavonoids, polyphenols, saponins, tannins, terpenoids, xanthenes, quinonoids, steroids, and alkaloids. These bind to the toxic proteins of the venom, thereby inactivating them. They could also competitively block target receptors [80]. Flavonoids have been reported for their ability to interact with the components of the snake venom and inhibit their activity; for example, pinostrobin, an isolated flavanone from the leaves of *Renealmia alpinia*, has been reported for its anti-hemorrhagic and antimyotoxic activities, along with its ability to neutralize the in vitro activities against the venom of *B. asper* [81].

### 6.2. Enzyme Inactivation/Inhibition 

Snake venom phospholipase A2 (PLA_2_), metalloproteases, and hyaluronidases are the key enzymes involved in snake venom toxicity [82]. Thus, inactivation of these enzymes is generally considered the fundamental step in the management of snakebites. Polyphenolic compounds such as tannins are specialized metabolites found in many plant species and have been shown to interact with the enzymes of the snake venom by non-specific binding proteins [83]. Several studies have concluded that the effect of polyphenolic compounds in snake venoms is due to the interaction between the enzymes of the venom and the hydroxyl groups (through hydrogen bonds) in these types of metabolites, resulting in the formation of a stable complex [84]. It has been reported that the high-resolution crystal structures of the two PLA_2_ complexes isolated from the venom of *Daboia russelli pulchella* and made with anisic acid and atropine [85] indicated that the networks of hydrophobic interactions and hydrogen bonds stabilized the positions at the substrate by binding to the enzyme. Significant interactions with His48 and Asp49 were observed in both complexes [86].

### 6.3. Chelation Activity

Plant extracts have compounds that bind to divalent metal ions, which are necessary for some enzymatic activities. Since proper coordination of metal ions is a prerequisite for the hydrolytic activities of PLA_2_ and metalloproteases, any metabolite that can weaken the enzyme–metal ion interaction will result in inactivation of the hydrolytic activity [87].

### 6.4. Adjuvant Action 

The 2-hydroxy-4-methoxybenzoic acid isolated from the root extract of *Hemidesmus indicus* neutralized the pathophysiological changes induced by snake venoms through adjuvant effects and potentiation of the antiserum. Increased production of antibodies in hyperimmunized rabbits was evidenced from the increased neutralization of venom (lethal and hemorrhagic activity). This compound also acted as an adjuvant by triggering the retention of small venom antigen particles and aiding in the formation of antibodies [78].

### 6.5. Antioxidant Activity

Compounds such as vitamins A, C and E, flavonoids, terpenoids, tannins, polyphenols, and some minerals (i.e., selenium) from plants have the ability to neutralize free radicals; hence, they are valuable natural antioxidants that can scavenge and remove oxygen free radicals, stabilize cell membranes, and act as immunomodulators [88]. These classes of compounds are known to be powerful antioxidants both in hydrophilic and lipophilic environments. They can prevent, stop, or reduce oxidative damage due to PLA_2_ activity by selectively binding to the active sites or modifying the conserved residues that are critical for the PLA_2_ catalysis [83].

## 7. Natural Products in the Development of Antivenom Agents

The term “natural products” spans an extremely large and diverse range of chemical compounds derived and isolated from biological sources such as plants, minerals, and organic matter. Interest in natural products that have been used for over a thousand years is continuing based on the experience of randomized trials and animal observations. In ancient times, people acquired knowledge on plant use to treat diseases. For example, Chinese herbal medicine (CHM) and Indian herbal medicine (Ayurvedic) were highly developed in antiquity. China, Japan, Korea, and India still influence modern healthcare [32]. In recent years, natural products have experienced a resurgence in drug discovery programs, mainly due to their superior chemical diversity over synthetic compound libraries and their drug-like properties. There are several widely used drugs derived from natural sources, which are available in the form of food supplements, nutraceuticals, and complementary and alternative medicines. In fact, some widely used drugs used to treat certain life-threatening diseases are derived from natural sources, such as paclitaxel and artemisinin, which are used as anticancer and antimalarial agents, respectively [38]. 

As already discussed in Section 11, people rely heavily on other therapeutic alternatives from traditional healers with knowledge based on ancient culture, ethnic practices, and herbal antidotes because of inadequate facilities and difficulties in timely medical care, especially in rural areas. The plant kingdom provides an inexhaustible source of various herbal compounds with pharmacological potential. A large number of medicinal plants are widely used by traditional healers in the form of baths, pastes, decoctions, powders, plasters, and pills for the treatment of snakebite envenomation [89,90]. It is a known fact that secondary metabolites isolated from different sources such as plants, fungi, and bacteria are natural, have no potential side effects, are stable for a long time, can be easily stored, and some can neutralize a wide range of snake enzymes, such as phospholipase A2 (PLA_2_), hyaluronidase, protease, L-amino acid oxidase, and 5′ nucleotidase, among others. Table 2 summarizes a list of bioactive compounds that have been studied, tested, and reported for the inhibition of one or more enzyme components of different venoms, both in in vitro and in vivo models. 

**Figure 2:**
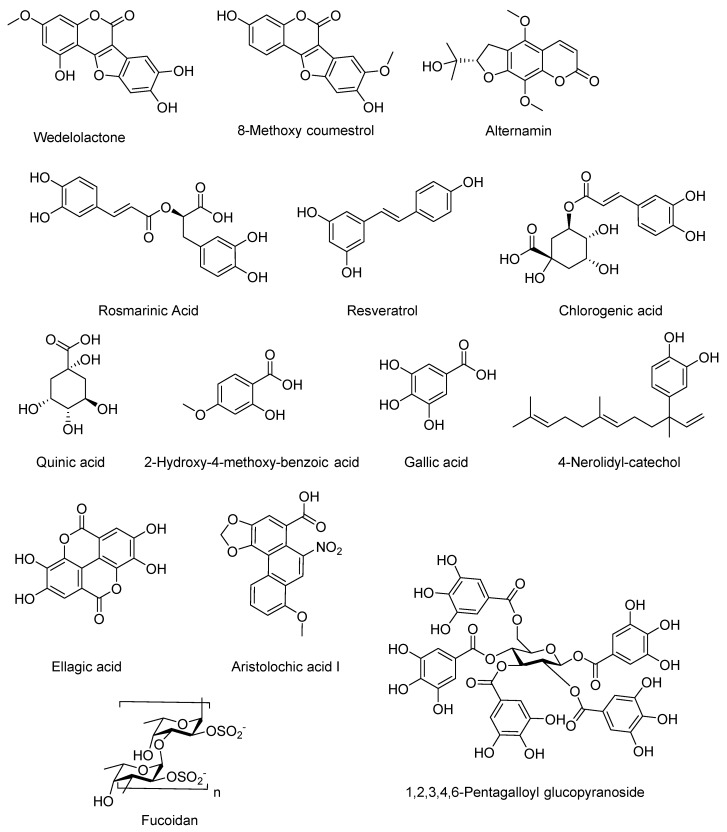
Selected phenolic compounds and the fucoidan sugar with antivenom activity.

The isolated small molecules with antivenom activity were categorized based on their chemical structures. Phenolic compounds are important components of plants and include hydroxybenzoic acids, coumarins, cinnamic acids, and polyphenolic compounds, among others. These essential plant constituents display antioxidant properties by trapping the reactive oxygenated species [91] and exhibit interaction with hydrolases, oxidoreductases, and isomerases [92]. Additionally, several phenolic compounds exhibit phospholipase A2 inhibition against numerous snake venoms [93,94,95]. There are a number of mechanisms through which phenolic compounds act as anti-snake venoms. These include the elimination of free radicals, hydrogen donation, singlet oxygen cooling, metal ion chelation, or as substrates during attack by superoxides. Another approach for the potent neutralization of snake venom by phenolic compounds could be due to the blocking of one or more enzymatic active sites in the venom toxins or the receptors that are structurally susceptible to chemical attack [93]. The aristolochic acid I (8-methoxy-6-nitro-phenanthro(3,4-d)1,3-dioxole 5-carboxylic acid) is an alkaloid present in *Aristolochia* species. The aristolochic acid I isolated from *Aristolochia radix* and *Aristolochia odoratissima* [96] inhibits enzymatic and pharmacological activities of a basic PLA_2_ from *V. russelli* venom [97]. In molecular coupling studies performed with aristolochic acid I and PLA_2_ isolated from *V. russelli* venom, [98] the most interesting interactions between these two substances are provided by their OH groups, which form two hydrogen bonds with His48 and Asp49. Moreover, according to Girish and Kemparaju [99], aristolochic acid I is an inhibitor of hyaluronidase of *Naja naja* venom. Hyaluronidase is one of the most widely distributed components of snake venom and has been characterized as a substance that aids in the spread of venom due to its ability to promote local hemorrhage. Consequently, the inhibition of hyaluronidase helps reduce both local tissue damage and the magnitude of the systemic effects of envenomation [100]. Figure 2 shows the selected phenolic compounds with antivenom activity.

**Figure 3:**
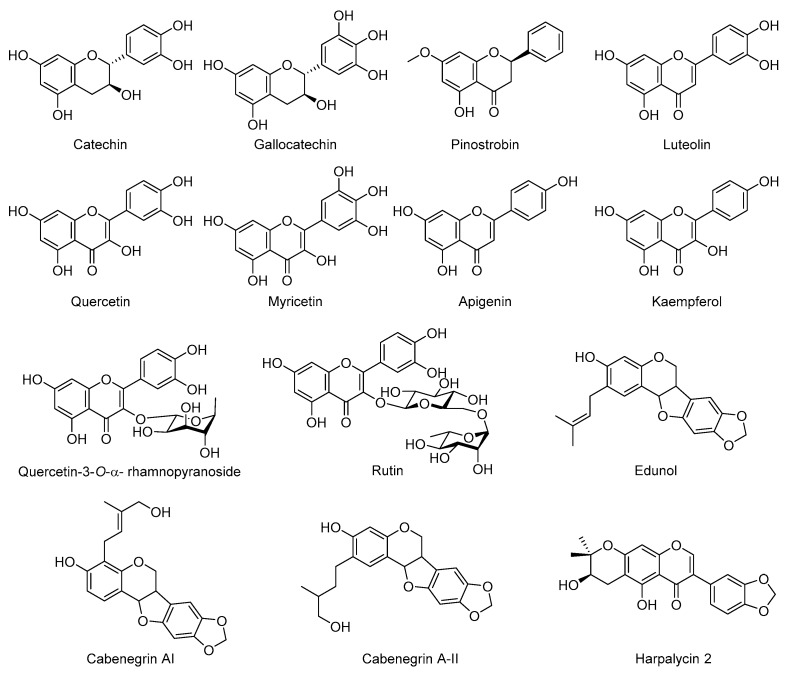
Selected flavonoids with antivenom activity.

Flavonoids are known for their wide range of health benefits and are commonly used in various ailments. The polypharmacological profile of flavonoids as anti-inflammatory, anti-mutagenic, and anti-carcinogenic agents is due to their ability to modulate key cellular enzyme functions; for example, xanthine oxidases, cyclooxygenases, and kinases [101]. It is because of these properties that flavonoids are active against several snake venoms, examples of which are shown in Figure 3. Dietary flavonoids, such as catechin, quercetin, luteolin, kaempferol, myrcetin, and apigenin, display high inhibition of venom hyaluronidases [102]. Reports show that pinostrobin is the bioactive component of *Renealmia alpinia* that inhibited the phospholipase A2 in *Crotalus durissimus* venom and displayed anti-hemorrhagic and analgesic activities in *Bothrops asper* snake venom [103]. Myricetin exhibited proteolytic and hemorrhagic activities along with the inhibition of the enzymatic activity of zinc metalloprotease of *B. atrox* venom [104]. Quercetin-3-*O*-rhamnoside has shown strong inhibition of phospholipase A2 and anti-hemorrhagic activity for *Naja naja* venom [105]. Recently, rutin, another well-known dietary flavonoid with antioxidant property, ameliorated coagulation disorders involved in *Bothrops jararaca* envenomation [106]. Isoflavonoids, a subclass of flavonoids, are frequently found in free form in a great variety of plants. Isoflavonoids have been reported to be active against the snake venoms, cabenegrins AI and A-II from *Annona coriacea* (Annonaceae). They are known to reverse the toxic effects of the *B. atrox* venom in dog models; however, no mechanism has been reported [107]. Edunol from *Brongniartia podalyrioides* (Leguminosae) reversed the toxic effects of *B. atrox* envenomation in rodent models [45]. Harpalycin 2 (HP-2) isolated from *Harpalyce brasiliana* (Papilionoideae) inhibited the enzymatic acitivity of piratoxin-III isolated from the venom of *Bothrops pirajai* [108]. 

**Figure 4:**
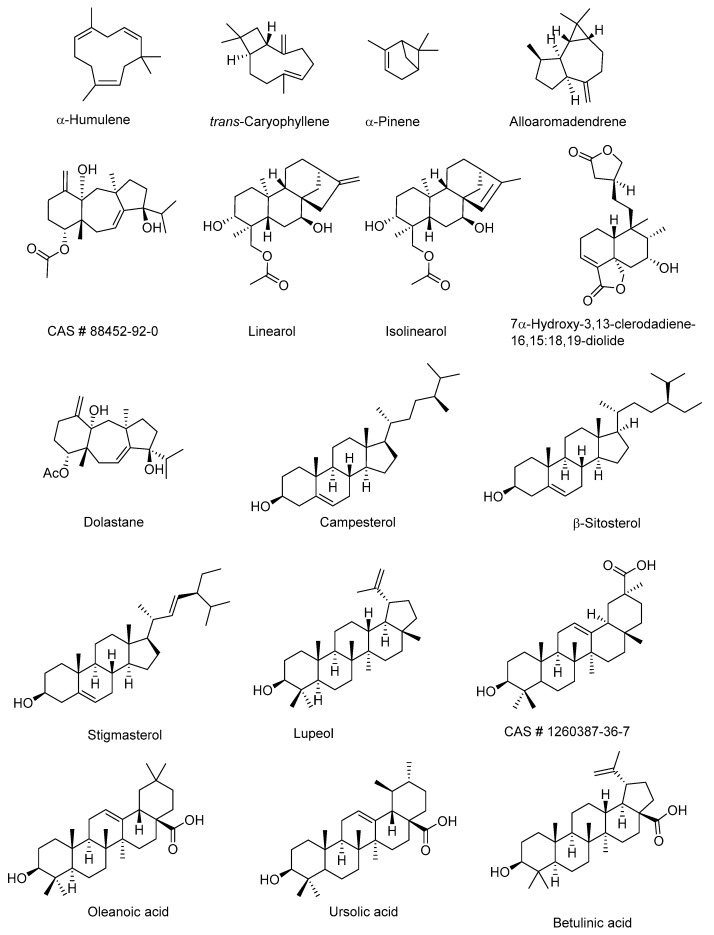
Selected terpenoids with antivenom activity.

**Figure 5:**
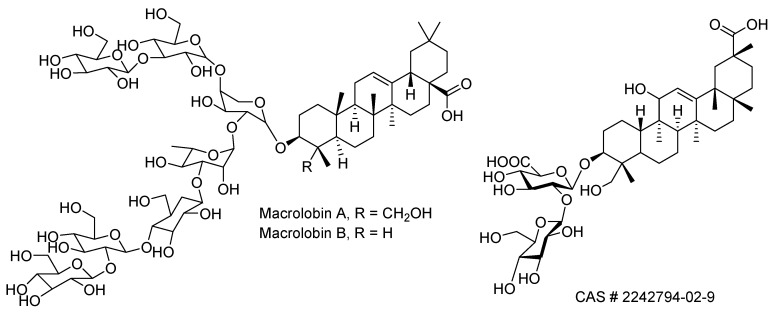
Selected saponins with antivenom activity.

Terpenes are a combination of isoprene units and display a wide range of biological properties and therapeutic uses [109]. There are several examples of monoterpenes, sesquiterpenes, diterpenes, triterpenoids, and steroids, and their glycosides that have been used to treat the effects of snakebites. Selected examples are shown in Table 2 and Figure 4. Linearol and isolinearol isolated from the brown algae *Canistrocarpus cervicornis* inhibited hemolysis, proteolysis, and hemorrhage in *Bothrops jararaca* envenomation [110]. Steroids such as sitosterol, stigmasterol, and campesterol, and pentacyclic triterpenoids such as lupeol showed anti-hemorrhagic and anti-lethality properties against several snake venoms due to their antioxidant properties [85]. Triterpene (CAS # 1260387-36-7) exhibited phospholipase A2 activity [111]. A typical example is the metalloproteinase activity displayed by the heptaglycoside saponins, macrolobins A and B (Figure 5) [112]. Betulinic, oleanoic, and ursolic acids have shown potent metalloproteinase inhibition and proteolytic activity for *Bothrops atrox* venom, which are evident from computational studies [113]. Dolastane, a diterpene isolated from the extract of the marine brown algae *Canistrocarpus cervicornis* (Dictyotaceae) was found to inhibit hemorrhage, hemolysis, and coagulation induced by the venom of *Lachesis muta* [114]. Oleanolic acid, a triterpenoid, known for its anti-inflammatory property, is commonly present in several medicinal plants. Oleanolic acid inhibited the activities of sPLA_2_ (a significant enzyme involved in inflammatory reactions) of human pleural fluid (HPF), human synovial fluid (HSF), *Vipera russelli*, and *Naja naja* snake venoms [115]. 

**Figure 6:**
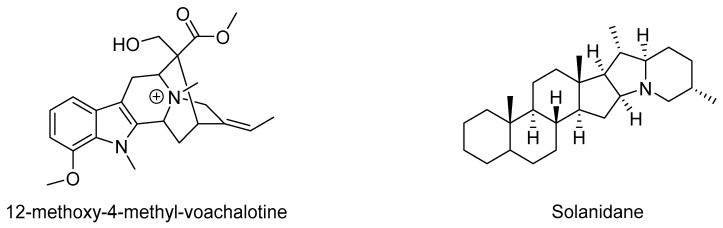
Selected alkaloids with antivenom activity.

There are several examples of alkaloids with antivenom properties (Table 2, Figure 6). In the year 2000, it was reported that the compound 12-methoxy-4-methyl voachalotine isolated from the roots of *Tabernaemontana catharinensis* was able to neutralize the lethality and myotoxicity induced by *Crotalus durissus* venom [116]. Lately, it was found that the latex of *T. catharinensis* has several other indole alkaloids that are potential candidates for screening against snake phospholipases and metalloproteases [117]. 

Table 2 summarizes a list of isolated bioactive compound and their mechanisms of action.

## 8. Drawbacks of Herbal Products in the Treatment of Snake Envenomation

In general, the practice of traditional medicine is largely based on the use of plant derivatives, which can be classified into two classes: herbs found in databases that provide a detailed description and proof for their use in historical reports (for example, herbs used in traditional Chinese medicine and Ayurvedic medicine), and herbs used in folk medicine that lack bibliographic information and are transmitted verbally. It is undeniable that the development of many drugs in modern medicine is still based on clinical experiences of traditional medicines and therapies. A unique example is China, with at least 130 new drugs currently in clinical use that are unique chemical entities extracted from either medicinal herbs or synthetically modified compounds [155]. Some examples are anisodamine [156], indirubin [157], huperzine [158], and bicyclol [159]. Currently, the development of drugs, especially antivenoms and phyto-antivenoms, face three major challenges: the amount of time consumed, money spent, and potential toxicity of the obtained products. In this regard, traditional knowledge and pragmatic databases derived from clinical practice are fundamental to increase the success rate of drug discovery compared to the conventional approaches, which include isolation, screening, and chemical synthesis, leading to longer times. According to Si-Yuan Pan et al. [30], some of these databases are: Traditional Chinese Medicine Information Database (TCHM-ID), Traditional Chinese Medicines Integrated Database (TCMID), The Herb Information Knowledge Base (THINKherb), and Indian Plant Anticancer Compounds database (InPACdb). Available databases in drug discovery include: Online Mendelian Inheritance in Man (OMIM), World Molecular Bioactivity (WOMBAT), Therapeutic Targets Database (TTD), PubChem, DrugBank, BioAssay, and Potential Drug Target Database (PDTD). 

According to Laustsen and Dorrestijn [160], there is a need to provide safe, affordable, and effective antivenoms to victims of snakebites worldwide. It is important to take advantage of new biotechnological approaches for the development of new antivenoms. These could be based on mixtures of human monoclonal antibodies that could directly replace the polyclonal equine antibodies that are currently being used in conventional antivenoms.

## 9. Challenges Involved in the Discovery of Antivenoms

The discovery of new antivenoms involves significant challenges in the assessment, design, and production of potential antivenoms, and the refinement of current compounds to better meet the needs. New and much improved antivenoms with high standards can be produced in adequate volumes when multidisciplinary, international collaborative efforts are undertaken. This would help serve not a particular nation but entire regions [161].

The initial strategy included developing antibodies in sheep rather than in horses to refine Fab fragments in order to hasten tissue distribution and reduce the risk of anaphylactic reactions. This strategy was discontinued due to rapid renal elimination of Fab fragments and the need for recurrent envenoming [162]. Proteomic investigation of snake venoms is another strategy that needs to be determined, characterized, and harnessed for bioactive toxins in drug discovery. Proteomics support not only the design improvement of both antivenoms and immunogen mixtures but also provide a mechanism to evaluate the suitability of both existing and potential antivenoms [162]. In order to simplify proteomes, removal of highly abundant proteins was commonplace, which was later discontinued due to the loss of numerous minor species along with target proteins [161]. 

The combinatorial peptide library approach utilizes the snake venom protocol to mine and complement the gained data for comprehensive visualization of the venom proteome [163,164,165]. Another approach is the peptide inhibitor approach. In order for this approach to be successful, the inhibitors are required to have smaller molecular sizes and should not be immunogenic in nature, which might cause cross-reaction with acetylcholine receptors (AChR), resulting in myasthenia gravis, an autoimmune disease [161]. The phage display library is an alternative approach for the identification of epitopes in several proteins and toxins to find peptides that can bind to toxins (mimotopes) or antibodies specific against toxins. The benefit of this approach is conformation and that the linear epitopes are recognized from their corresponding mimotopes [161,166]. Synthetic biology is another approach to increase the production of antivenoms as compared to conventional methods. This approach involves the injection of small pieces of synthetic venom antibodies that are synthesized in *Escherichia coli* to boost the animal’s immune response. The advantage with synthetic biology is that the antivenoms produced through this approach result in less muscle damage and tissue death at the site of the bite, as these antibodies are smaller and better able to penetrate the tissue [165]. Development of a single optimized nanoparticle (NP) formulation led to the possibility of broad spectrum neutralization and sequestration of venomous biomacromolecules. This led to the possibility of broad spectrum antivenoms. The major challenge with this approach is the selectivity for the targeted venom proteins over other copious serum proteins when developing the polymer sequestrant [167].

## 10. Snake Venom as a Source of Therapeutic Agents

Although snake venom is fatal in itself, it is known to possess various toxin peptides with significant bioactivities [22,168]. Tirofiban and eptifibatide are FDA-approved antiplatelet drugs, which are disintegrin derivatives from *Echis carinatus* and *Sistrurus miliarus barbouri*, respectively [169]. Captopril is a FDA-approved antihypertensive drug, which is a derivative of bradykinin potentiating peptide obtained from *Bothrops jaracusa* [170]. Hemocoagulase and batroxobin are marketed drugs in some countries outside of the United States for the treatment of hemorrhage and as a defibrinogenating agent, respectively. Hemocoagulases are thrombin- and thromboplastin-like enzymes obtained from *Bothrops atrox*, while batroxobin is a serine protease derived from *Bothrops moojeni* and *B. atrox*. Batroxobin can be a potential tool in patients on anticoagulant therapy for surgical hemostasis [171]. Ximelagatran is a peptide isolated from cobras, which was once a FDA-approved drug as an anticoagulant, but is now withdrawn from the market. Ancrod was also an FDA-approved drug as a defibrinogenating agent, which was later withdrawn and is currently under phase III clinical studies. Ancrod is an enzyme from *Agkistrodon rhodostoma*. Dendroaspis-NP is currently under phase II clinical study for the treatment of congestive cardiac failure. It is isolated from *Dendroaspis angusticeps* and is a natriuretic peptide. Furthermore, α-Cobratoxin and α-cobrotoxin are neurotoxins isolated from *Naja kaouthia* and *Naja nivea*, respectively. These neurotoxins are currently undergoing human trials as analgesics in China [172].

The two crucial New Guinean species used in the production of anti-venom are *Oxyuranus scutellatus* and *Acanthophis laevis*. Major species of South and Southeast Asian snakes used in antivenom production include *Calloselasma rhodostoma*, *Echis carinatus*, *Naja spp*., *Daboia spp*., *Bungarus spp*., and *Cryptelytrops spp.* In Africa, species belonging to *Cerastes*, *Dendroaspis*, *Naja*, *Bitis*, *and Echis* genera are significant for antivenom production [161].

Venom is a mixture of peptides, proteins, and other small molecules with varied pharmacological properties. There are many bioactive venom peptides from undiscovered sources that could result in potentially new therapeutic leads. The low approval rate of venom peptides could be due to problems such as the low stability and bioavailability of these peptides. Introduction of peptidomimetics into the organic compounds could be a solution to overcome the above problems by mimicking the peptide action [172].

## 11. Conclusions

Natural products are biodiverse in nature and can be invaluable resources that can contribute to the continuous improvement of the development of products that act as co-adjuvants and can help neutralize the action of venom toxins. As drug discovery from natural sources has traditionally been time-consuming, faster and better methodologies for bioassay screening, compound isolation, and compound development must be employed. Even with all the limitations facing drug discovery, natural products isolated from medicinal plants remain as the essential tools in the search for new medicines. Although there are many studies with clear evidence of the effectiveness of herbal treatments for snake bites, very few of these studies have clinical corroboration. Therefore, it is important that the use of such plants for the treatment of snake bites should be performed with caution until efficiency can be confirmed. In this review, we focused on the collection of data from isolated plants and compounds that are most commonly used in the treatment of snakebites. The continuation of research in this area is urgent, especially in rural areas of countries that have received less attention. Novel approaches should be recognized for the identification of active ingredients to treat snakebites, and the continuum of the virtual methods can expedite the process though analytical chemistry tools. With thousands of plant species on the planet, there is a wide diversity of sources to obtain medicinal remedies from nature. Due to the low success rate and the huge need for capital investment, the research and development of conventional drugs is very expensive and difficult to achieve. Consequently, researchers have focused their efforts on discovering drugs from natural sources. The exceptional knowledge transmitted by indigenous and rural communities regarding the use of plants for the treatment of various diseases should be used to improve the success rate of the development of new drugs or phytotherapeutics. Since this process is initially based on experience, and therefore has a known approach, the search for therapeutically useful synthetic drugs implies greater difficulty and is a daunting task. The use of synthetic biology has appeared to revolutionize drug discovery, and hence there are fewer success stories without serious and unpredictable complications. 

We are living in a post-genomic era, and therefore, the prospect of the drug discovery from herbal medicines and other organisms is growing. Ongoing efforts involve the use of advancements in science and technology for the development of new and better processes that could ultimately lead to novel and efficient drugs. Pharmaceutical companies face a great challenge in exploring new forms of research and development in drug discovery, and hence, it is necessary that they become cognizant of both herbal medicines and other sources of bioactive compounds. Small molecules with different natural scaffolds have the potential to act as a single entity or as a mixture to affect the modulation of the toxic enzymes present in snake venoms. Increased research towards the development of bioactive natural products as lead compounds to overcome the need for novel antivenoms is essential.

## Figures and Tables

**Table 1:** Research and ethnopharmacological studies from different countries for the treatment of snake envenomation.

Country	Plant	Description of the Study	Reference
India	*Morus alba* (Moraceae)	Extracts from *Morus alba* (Moraceae) are active against *Daboia russelli* venom, inhibiting the caseinolytic, hyaluronolytic, edematogenic, hemorrhagic, and procoagulant activities.	[47]
Nigeria	*Mucuna pruriens* (Fabaceae)	Seed extract of *Mucuna pruriens* (Fabaceae) used in Nigerian communities offer significant protection to cardiac muscle tissue and blood vessels, and even protects against the lethality produced by venoms from *Naja kaouthia*, *Naja nivea*, and *Calloselasma rhodostoma*. This protection can be explained from the presence of a Kunitz-type trypsin inhibitor.	[48,49,50]
Yemen	*Hibiscus aethiopicus* (Malvaceae)	Aqueous crude extracts from *Hibiscus aethiopicus* (Malvaceae) possess significant anti-hemorrhagic and cytoprotective activities against *Echis ocellatus* and *Naja n. nigricollis* venoms	[51]
India	*Vitis vinifera* (Vitaceae)	Administration of the methanolic extracts from *Vitis vinifera* (Vitaceae) resulted in the reduction of local symptoms produced by *D. russelli* venom due to the inhibition of the proteolytic and hyaluronidase activities reducing edema, myonecrosis, and hemorrhaging.	[52]
Brazil	*Dipteryx alata* (Fabaceae)	Extracts and fractions from *Dipteryx alata* (Fabaceae) partially neutralized *Bothrops jararacussu* and *Crotalus durissus* terrificus venom activities. Hydroalcoholic bark extract from *D. alata* is active against *B. jararacussu* venom.	[53,54]
Brazil	*Marsypianthes chamaedrys* (Lamiaceae)	Infusions and crushed leaves from *Marsypianthes chamaedrys* (Lamiaceae) showed a similar activity produced by antivenom serum against clotting and inflammatory effects of the *Bothrops atrox* venom	[55]
Brazil	*Hypericum brasiliense* (Guttiferae)	*Hypericum brasiliense* (Guttiferae) reduces the lethality produced by *Bothrops jararaca* by inhibiting the edematous and proteolytic activities of the venom.	[56]
India	*Luffa egyptiaca* (Cucurbitaceae) and Nicotiana rustica (Solanaceae)	Ethyl acetate fractions of *Luffa egyptiaca* (Cucurbitaceae) and *Nicotiana rustica* (Solanaceae) extracts completely inhibited the protease activity of *Naja nigricollis* venom.	[57]
India	*Mangifera indica* (Anacardiaceae)	Aqueous extracts from the stem bark of *Mangifera indica* (Anacardiaceae) inhibited the protease, hyaluronidase, hemorrhagic, fibrinogenolytic, hemolytic, procoagulant, edema, ATPase, and alkaline phosphatase activities produced by *D. russelli* venom	[58]
Argentina	*Nectandra angustifolia* (Lauraceae)	Ethanolic extracts and essential oils from *Nectandra angustifolia* (Lauraceae) leaves inhibited the hemolytic and coagulant effects produced by *Bothrops neuwiedi* venom	[59]
Pakistan	*Fagonia cretica* (Zygophyllaceae)	Methanolic extract from leaves and twigs of *Fagonia cretica* (Zygophyllaceae) is capable of inhibiting hemorrhage induced by *Naja naja karachiensis* venom	[60]
Brazil	*Sapindus saponaria* (Sapindaceae)	Fractions of the hydro alcoholic extracts from the callus of *Sapindus saponaria* (Sapindaceae) partially inhibited the lethality, phospholipase, clotting, edema, hemorrhagic, and myotoxic activities produced by *Bothrops jararacussu*, *Bothrops moojeni*, *Bothrops alternates*, and Crotalus durissus terrificus venoms along with isolated myotoxins and phospholipase A2 (PLA_2_)	[61]
Brazil	*Mouriri pusa* (Melastomataceae), *Byrsonima crassa* (Malpighiaceae), and *Davilla elliptica* (Dilleniaceae)	Methanolic extracts from *Mouriri pusa* (Melastomataceae), *Byrsonima crassa* (Malpighiaceae), and *Davilla elliptica* (Dilleniaceae) blocked local hemorrhages produced by *Bothrops jararaca* venom.	[62]
India	*Anacardium occidentale*	The efficacy of *Anacardium occidentale* extract against pharmacological actions induced by *Vipera russelli* venom was observed from the neutralization of phospholipases, proteases, and hyaluronidases, as well as edema, hemorrhage, lethality, and myonecrosis effects	[63]
India	*Tamarindus indica* (Fabaceae)	*Tamarindus indica* (Fabaceae) inhibited hyaluronidase, Phospholipase A2 (PLA_2_), l-amino oxidase (LAAO), and 5′-nucleotidase. Exhibited fibrinogenolytic, edema-inducing, hemorrhagic, indirect hemolytic, coagulant, and myotoxic properties, and protected against venom-toxicity	[64]
Brazil	83 plant species, from 34 families	Inhibition of Phospholipase A2 (PLA_2_), anti edema, anti lethality, anti clotting, myotoxicity, and antihaemorrhagic activity	[65]
Brazil	*Mandevilla velutina and Eclipta prostata*	Inhibition of creatine kinase release and myotoxic activity	[66]
Worldwide	*Schumanniophyton magnificum, Aristolochia radix, Diospyros kaki, Alocasia cucullata, Picrasma quassioides, Eclipta prostrata, Curcuma sp., Soja hispida, Diodia scandens, Andrographis paniculata*	Inhibition of Phospholipase A2 (PLA_2_) or other enzymes (Adenosine triphosphatese), and life-prolongation effect post black mamba venom treatment.	[67]
Colombia	77 plant species	Three relevant studies: First study was an inventory with 77 species of plants belonging to 41 families used by Colombian healers along with the methods of preparation, administration, and dosage; second study was a list of 74 ethanolic plant extracts used by folk medicinethat were active against lethal effects produced by *Bothrops atrox* venom; third study showed 31 extracts with moderate or high neutralizing abilities against the hemorrhagic effect of *B. atrox* venom	[68]
Brazil	Numerous plant species	Review discussing Brazilian plant species displaying neutralizing properties against snake envenomation from an ethnopharmacological perspective	[69]
Costa Rica	40 plant species	40 plant species belonging to at least 30 families. Neutralization activity of Costa Rican plants towards *B. asper* venom and toxins	[70]
India	34 plant species	A list of 34 plant species belonging to the Zingiberaceae family traditionally used in Northeast India, where one species presented antivenom activity and five other species have been scientifically validated to be anti-inflammatory	[71]
Nepal, China, South Africa, Nicaragua, and Brazil	310 plant genera from 171 families	Enzyme inhibition activity	[72]
India	69 plant species	69 plant species belonging to 29 genera and 17 compounds with antiophidian activity or relative properties against venoms from 34 snake species	[73]
Nigeria and Ghana	*Schumanniophyton magnificum, Strophanthus gratus, Strophanthus hispidus, Mucuna pruriens*	Aqueous extracts showed effects on the blood clotting against *Echis carinatus* envenomation	[74]
Mali, DR Congo, South Africa	94 species of 84 genera	List of plants used traditionally in sub-Saharan Africa. Hyaluronidase, phospholipase A2, and protease inhibitory activity against effects produced by *Bitis arietans*, and *Naja nigricollis* venom	[75]

**Table 2:** List of isolated bioactive compounds with venom neutralization capabilities and their mechanisms of action.

Compound	Source	Mechanism of Action	Mode of Administration/Study Level	Reference
Aristolochic acids	*Aristolochia indica* *Aristolochia sp.* *Aristolochia odoratissima* *Aristolochia fordiana* *Aristolochia radix*	Induction of PLA_2_ Inhibition of L-amino acid oxidase Anti-lethality	Injected into the mouse foot pad	[96,97,98,99,118,119,120,121]
Rosmarinic acid	*Cordia verbenacea* *Argusia argentea*	Inhibition of myotoxic activity and PLA_2_ Anti-hemorrhagic activity	Injected intramuscularly into the right gastrocnemius muscle of mice In vivo study	[122,123,124,125]
Quercetin-3-O-α-L-rhamnopyranoside quinic acid, gallic acid, quercetin, kaempferol, luteolin, ellagic acid, chlorogenic acid	*Euphorbia hirta*	Inhibition of PLA_2_	In vitro study	[105,126]
Pinostrobin	*Renealmia alpinia*	Inhibition of myotoxic activity and PLA_2_	Intramuscular injection (inhibition of myotoxic activity) and subcutaneous injection (inhibition of edema-inducing activity)	[81]
Undisclosed	*Azadirachta indica*	Inhibition of PLA_2_	In vitro study	[127]
2-hydroxy-4-methoxy benzoic acid	*Hemidesmus indicus*	Neutralization of venom hemorrhagic activity	Injected intradermally into mice	[128]
β-sitosterol	*Eclipta prostrata, Humirianthera ampla* *Cynanchum paniculatum*	Neutralization of enzymes	In vitro and in vivo studies	[129,130,131]
8-methoxy coumestrol	*Medicago sativa*	Inhibition of edema, hemorrhage, and cardio toxicity	Injected intravenously into mice	[132]
7α-hydroxy-3,13-clerodadiene-16,15:18,19-diolides	*Baccharis trimera*	Inhibition of metalloproteases	In vitro and in vivo studies	[133]
CAS # 1260387-36-7 CAS # 2242794-02-9	*Clematis gouriana*	Inhibition of PLA_2_	In vitro and computational studies	[111]
Linearol Isolinearol CAS # 88452-92-0	*Canistrocarpus cervicornis*	Inhibition of induced hemorrhage, hemolysis, and coagulation	Biological assays	[110,114]
Ellagic acid	*Casearia silvestris*	Anti-myotoxic and anti-edema	In vivo study	[134,135]
Resveratrol	*Crinum jagus*	Neutralization of PLA_2_, protease, hyaluronidase, L-amino acid oxidase, and 5′-nucleotidase enzyme activities. Anti-myonecrosis and anti-hemorrhagic	Oral and intraperitoneal administration. In vivo study	[136,137]
Campesterol, β-sitosterol (its glycoside), stigmasterol, catechin, and gallocatechin	*Croton urucurana*	Anti-hemorrhagic and anti-lethality	In vivo study	[138]
Rosmarinic acid α-Humulene (−)-*trans*-Caryophyllene α-Pinene and alloaromadendrene	*Cordia verbenacea*	Anti-inflammatory, anti-myotoxic, anti-edematogenic, and anti-PLA_2_ activity. Anti-edematogenic and reduction of tumor necrosis factor-α (TNFα) Reduction of platelet activating factor, bradykinin, and anti-edematogenic. Anti-inflammatory and anti-edematogenic	In vivo study	[87,122,139,140]
Fucoidan	*Fucus vesiculosus*	Anti-myotoxic activity, anti-PLA_2_, and anti-necrosis	In vivo study	[141]
Wedelolactone	*Eclipta alba*	Anti-hepatotoxic, anti-hypertensive, anti-tumor, anti-PLA_2_, anti-snake venom, and anti-myotoxic-induced PLA_2_.	In vivo study	[142,143,144]
Wedelolactone, sitosterol, and stigmasterol	*Eclipta prostrata*	Anti-neurotoxic and anti-myotoxic	In vivo study	[145]
2-hydroxy-4-methoxy-benzoic acid and lupeol acetate	*Hemidesmus indicus*	Anti-defibrinogenatic, anti-edematogenic, anti-PLA_2_ activity, anti-necrotic, anti-hemorrhagic, anti-coagulant, lipid peroxidase inhibition, superoxide dismutase activity, antiserum action potentiation, anti-lethality, anti-cardiotoxic, and anti-neurotoxic	In vivo study	[78,79,128,146]
Pentagalloyl glucopyranose	*Mangifera indica*	Anti-hemorrhagic, anti-dermonecrotic, and enzymatic activities. Inhibition of protease, hyalunoridase, fibrinogenolytic, procoagulant, anti-edematogenic, anti-ATPase, and alkaline phosphatase	In vivo study	[60,83]
Gallic acid	*Musa paradisiaca*	Anti-PLA_2_, anti-myotoxic, anti-hemorrhagic, and anti-lethality	In vivo study	[109,147]
Alternamin	*Murraya alternans*	Anti-hemorrhagic	In vivo study	[148]
Macrolobins A and B	*Pentaclethra macroloba*	Anti-proteolytic and anti-hemorrhagic, metalloprotease inhibitors	In vitro and in vivo studies	[112]
4-Nerolidyl-catechol	*Piper umbellatum and* *Piper peltatum*	Anti-myotoxic, anti-PLA_2_, anti-serineprotease, and anti-edematogenic	In vivo study	[42]
Solanidane	*Solanum campaniforme*	Hemorrhagic inhibitor, necrotizing, and myotoxicity effects	In vivo study	[149]
12-methoxy-4-methyl-voachalotine	*Tabernaemontana catharinensis*	Inhibited lethality	In vivo study	[116,117]
*Whitania Somnifera* Glycoprotein (WSG)	*Withania somnifera*	Anti-edematogenic	In vivo study	[150]
β-sitosterol, quercetin-3-O-glucopyranoside, and kaempferol-3-O-glucopyranoside	*Morus nigra*	Anti-inflammatory and antinociceptive effects	In vivo study	[151]
2-hydroxy-3-methoxy benzaldehyde	*Janakia arayalpathra*	Anti-PLA_2_	In vitro study	[92]
Marmin	*Aegle marmelos*	Anti-snake venom	In vivo study	[85]
Cabenegrins AI and A-II	*Annona coriaceae*	Anti-snake venom	In vitro and in vivo studies	[107]
Boc-5 and Boc-10 (sulfated galactans)	*Botryocladia occidentalis*	Anti-edematogenic, anti-myotoxic, and anti-neurotoxic	In vivo study	[152]
Edunol	*Brongniartia podalyrioides*	Protective effect against *Bothrops atrox* venom	In vivo study	[45]
Dolastane	*Canistrocarpus cervicornis*	Inhibition of induced hemorrhaging, hemolysis, and coagulation	In vitro and in vivo studies	[116]
Manoalide	*Luffariella variabilis*	Inhibition of extracellular PLA_2_ activity of cobras	In vitro study	[153]
DM64 (acidic glycoprotein)	*Didelphis marsupialis*	PLA_2_ inhibitor and prevention of myofiber breakdown caused by myotoxins I (Asp49) and II (Lys49) of *B. asper* venom	In vitro and in vivo studies	[154]
Aristolochic acid (8-methoxy-6-nitrophenanthro(3,4-d)-1,3-dioxole-5-carboxylic acid) Caffeic acid (3-(3,4-dihydroxyphenyl)-2-propenoic acid)	*Aristolochia species* *Vernonia condensate*	Inhibition of piratoxin-1 (PrTX-1), a Lys49-PLA_2_ isolated from *Bothrops pirajai* venom Inhibition of PrTX-1 and antidote activity against *B. jararaca* venom	Oral or parenteral administration In vitro and in vivo studies	[85,119] [121]
